# Real-Time Person Detection in Wooded Areas Using Thermal Images from an Aerial Perspective

**DOI:** 10.3390/s23229216

**Published:** 2023-11-16

**Authors:** Oscar Ramírez-Ayala, Iván González-Hernández, Sergio Salazar, Jonathan Flores, Rogelio Lozano

**Affiliations:** Aerial and Submarine Autonomous Navigation Systems Program, Cinvestav, Mexico City 07360, Mexico; oscar.ramirez@cinvestav.mx (O.R.-A.); ivan.gonzalez@cinvestav.mx (I.G.-H.); sesalazar@cinvestav.mx (S.S.); jonathan.flores@cinvestav.mx (J.F.)

**Keywords:** UAV, CNN, robust control

## Abstract

Detecting people in images and videos captured from an aerial platform in wooded areas for search and rescue operations is a current problem. Detection is difficult due to the relatively small dimensions of the person captured by the sensor in relation to the environment. The environment can generate occlusion, complicating the timely detection of people. There are currently numerous RGB image datasets available that are used for person detection tasks in urban and wooded areas and consider the general characteristics of a person, like size, shape, and height, without considering the occlusion of the object of interest. The present research work focuses on developing a thermal image dataset, which considers the occlusion situation to develop CNN convolutional deep learning models to perform detection tasks in real-time from an aerial perspective using altitude control in a quadcopter prototype. Extended models are proposed considering the occlusion of the person, in conjunction with a thermal sensor, which allows for highlighting the desired characteristics of the occluded person.

## 1. Introduction

Unmanned Aerial Vehicles (UAVs) are applied in many fields. UAVs have many advantages compared to ground vehicles [1], such as more degrees of freedom to avoid obstacles, coverage of a wide area in less time [2], and the detection of small objects, improving inspection coverage [3]. Object detection is one of the main machine vision applications used by UAVs. This task includes search and rescue missions in areas that are difficult to access, wide search areas, or areas affected by natural disasters [4]. Quick and planned actions are crucial to save as many lives as possible. Detecting people from aerial platforms has become an important aspect of the deployment of autonomous unmanned aerial vehicle systems in search and rescue missions. UAV systems have increased in popularity in various civil and scientific applications due to their ability to operate over large areas and difficult terrain [5,6].

In [7], the authors presented a robust automatic person-detection algorithm for search missions. These missions are a challenge due to the occlusion and radiation of trees in direct sunlight scenarios. The sunlight temperature reflected on the tree surfaces is similar to body temperature captured by camera sensors. Therefore, bodies in thermal images are poorly detected if they are partially hidden by trees. A simple threshold for the heat signal will not be appropriate for the detection of people [8].

In such situations, autonomous location reports for the detected objects of interest or human detection and recognition of bodies can eliminate the need for manual analysis of live UAV video images [9,10,11].

The image variation caused by movements and aerial vehicle instability generates blurred images. The UAVs’ attitude changes the visual shape of the captured object, affecting the size and position of the target object. These visual changes transform the appearance of the object, making detection harder [12].

**Figure 1:**
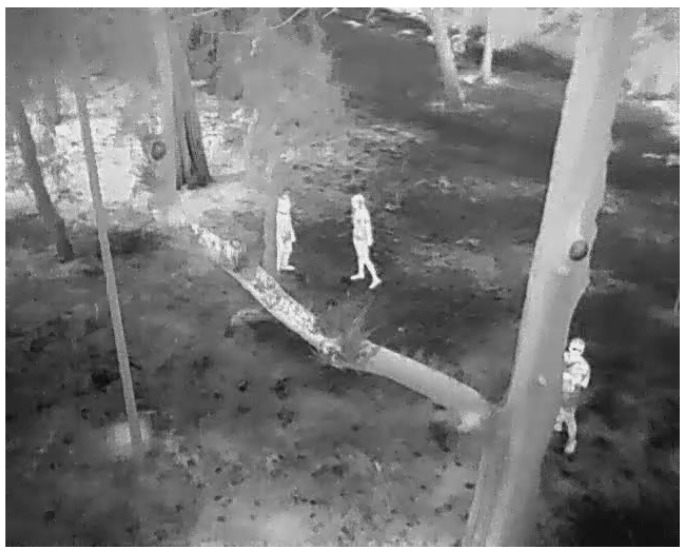
People aerial thermal image.

In critical situations, like forest fire monitoring and fighting, the use of UAVs has increased [13,14]. For example, in [15], a perception algorithm implemented in a UAV was presented to perform surveillance tasks using RGB aerial and thermal sensors when monitoring a specific area, see Figure 1.

Sliding Mode Control (SMC) was developed by Utkin in [16]. Its main contribution is to guarantee that a restriction on the sliding surface is satisfied. After verifying the restriction, the system trajectories converge in finite-time on the sliding surface. The advantages of SMC are the simplicity and robustness of the control strategy after choosing the sliding variable [17]. Fixed-time stability was introduced by Polyakov in [18]. Fixed-time stability ensures that the establishment time does not depend on the initial conditions and provides a predefined convergence time. Fixed-time stabilization with independent time controllers was proposed in [19,20,21].

## 2. Related Work

The present paper describes an approach to real-time person detection from thermal images captured from an aerial perspective in complex forest scenarios [22]. There are many challenges due the fast movements of the UAV, the image instability, and the relatively small size of the target person. Due to the altitude and attitude of the flight of the vehicle, the appearance of observed objects is complex, and it is difficult to detect and control the stabilization in real-time during the inspection of wooded areas with aerial vehicles.

In this work, the proposed CNN architecture analyzes images at a rate of six frames per second (fps) in order to maximize the image quality available for detection considering the embedded hardware limitations. Therefore, the proposed CNN + Haar model focuses on robust human detection within each individual captured frame [23].

To carry out human detection, sets of thermal images of people and a wooded area without people were formed. There are very few databases of this type, and so, the aim of the present work was to provide a new database that allows the improvement of the characteristics and detection conditions for deep learning algorithms when detecting partially occluded people. For the classification task, a Convolutional Neural Network (CNN) was used. The CNN is a deep learning technique that has been used successfully to solve problems related to computer vision [24].

Person detection presents a complex problem due to the small size of the target, the occlusion of people, and the low contrast of the human with respect to the background (Figure 1). Thermal imaging is used to reduce the optical camouflage present in the image. However, within thermal images, the detection of human traces remains a challenging problem due to the variability in these thermal signatures, generated by changing weather conditions, the thermography of the ambient environment, occlusion, or light-generated noise in the image. The main challenge is the dynamic and robust detection of people in different environments, both in clear and cluttered aerial views, regardless of the location or weather conditions.

We propose a real-time detection approach for detecting people using thermal images with the analysis of the thermal signatures.

The contributions are a database of thermal images of people and a compact CNN architecture assisted by a Haar cascade classifier for real-time applications, allowing the CNN model to be evaluated in an embedded computer from an aerial perspective. In order to obtain real-time images, a quadrotor aircraft was used. The hover flight was stabilized by a fixed-time sliding mode controller to compensate for unmodeled dynamics and external perturbations.

## 3. Quadrotor Aircraft Dynamical Model

**Figure 2:**
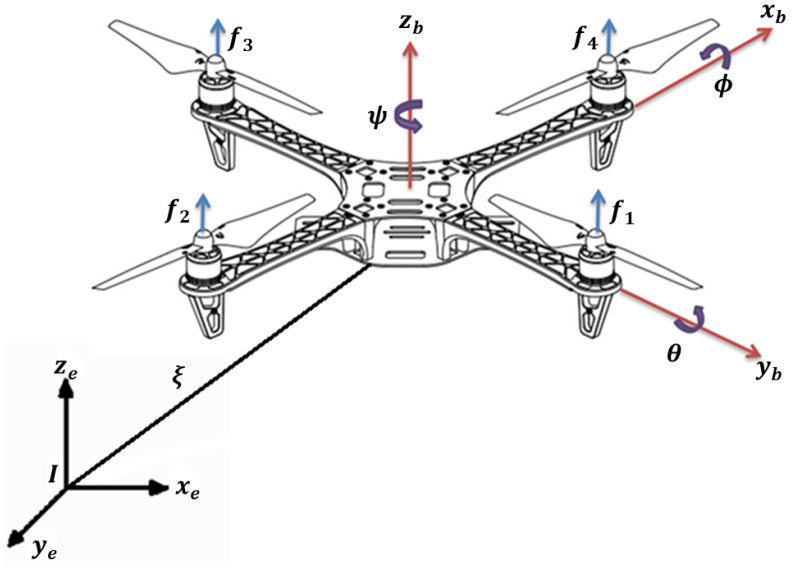
Free-body diagram of the quadrotor aircraft.

Quadrotors are under actuated systems with four control inputs and six degrees of freedom, which evolve in three-dimensional space. Their dynamical model makes them a system of interest to the scientific community because they contain under-actuated, strongly coupled, and multi-variable nonlinear dynamics. The aerial vehicle is considered as a rigid body that evolves in three dimensions, subject to a main force *u* generated by the propulsion of the rotors. The dynamical model of the drone is obtained from the Euler–Lagrange approach. Figure 2 shows a free-body diagram of the quadrotor aircraft.

The vehicle center of mass position with respect to the inertial frame *I* is denoted by

(1) $$\xi =\left[x,y,z\right]\in R^{3}$$

where *x* and *y* are the coordinates in the horizontal plane and *z* is the vertical position. The Euler angles are represented by

(2) $$\eta =\left[\phi ,\theta ,\psi \right]\in R^{3}$$

where ϕ is the roll angle around the *x*-axis, θ is the pitch angle around the *y*-axis, and ψ is the yaw angle around the *z*-axis; see Figure 2. The generalized coordinates of the vehicle are given by

(3) $$q=\left[x,y,z,\phi ,\theta ,\psi \right]\in R^{6}$$

The quadrotor dynamical model is obtained from the Euler–Lagrange methodology, and the equations can be divided into translational and rotational displacements.

The dynamical model of the quadrotor vehicle is

(4) $$m\ddot{x}=u\left(\cos\phi \sin\theta \cos\psi +\sin\phi \sin\psi \right)+d_{x}$$

(5) $$m\ddot{y}=u\left(\cos\phi \sin\theta \sin\psi -\sin\phi \cos\psi \right)+d_{y}$$

(6) $$m\ddot{z}=u\cos\theta \cos\phi -mg+d_{z}$$

(7) $$\ddot{\phi }=\dot{\theta }\dot{\psi }\frac{I_{y}-I_{z}}{I_{x}}+\frac{l}{I_{x}}{\tilde{\tau }}_{\phi }+{\tilde{d}}_{\phi }$$

(8) $$\ddot{\theta }=\dot{\phi }\dot{\psi }\frac{I_{x}-I_{z}}{I_{y}}+\frac{l}{I_{y}}{\tilde{\tau }}_{\theta }+{\tilde{d}}_{\theta }$$

(9) $$\ddot{\psi }=\dot{\phi }\dot{\theta }\frac{I_{y}-I_{x}}{I_{z}}+\frac{l}{I_{z}}{\tilde{\tau }}_{\psi }+{\tilde{d}}_{\psi }$$

where *m* is the mass and *l* is the distance from the center of mass to each rotor. $I_{x}$, $I_{y}$, and $I_{z}$ are the inertial constants in each axis. ${\tilde{\tau }}_{\phi }$, ${\tilde{\tau }}_{\theta }$, and ${\tilde{\tau }}_{\psi }$ represent the roll, pitch, and yaw torque, respectively. ${\tilde{d}}_{\phi }$, ${\tilde{d}}_{\theta }$, and ${\tilde{d}}_{\psi }$ represent the external perturbations, and $d_{x}$, $d_{y}$, and $d_{z}$ represent non-modeling, coupling, and external perturbations.

## 4. Control Based on the SMC Algorithm in Fixed-Time

For the implementation of the controller by sliding modes in fixed-time, we performed simulations to verify the behavior of the dynamics of the quadrotor. However, for real-time applications, there are disturbances such as wind gusts and unmodeled dynamics; it is necessary to use nonlinear control, which provides robustness [25,26]. A well-known control method is the Sliding Mode Control (SMC), whose strengths are the robustness in the face of the unmodeled dynamics of the system, which is essential in the search and rescue application.

### 4.1. Control Design

The objective is to control the altitude by means of the force *u*, and the movement on the $X_{b}$- and $Y_{b}$-axes is controlled by means of the desired angles θ and ϕ since it is not possible to apply forces directly in the $X_{b}$ and $Y_{b}$ directions with the rotors. The desired angles for the quadrotor should be defined, so that the force *u* generates components in the $X_{b}$ and $Y_{b}$ directions. The design problem is to enforce the behavior of the states towards the desired trajectory. The following procedure describes how to determine the control law for any of the dynamics of the quadrotor (*x*, *y*, *z*, ψ, θ, ϕ).

The translational dynamics of the quadrotor is defined in Equations (4)–(6); considering the dynamics in *z*, the vertical thrust force *u* is proposed as

(10) $$u=\frac{m(\nu_{z}+g)}{\cos\theta \cos\phi }$$

where $\nu_{z}$ is an auxiliary control that will be defined later. Introducing (10) into (6) the translational dynamics in the *z*-axis, we have

(11) $$\ddot{z}=\nu_{z}+d_{z}$$

Introducing (10) into the $\ddot{x}$ and $\ddot{y}$ dynamics, it follows that

(12) $$\ddot{x}=\left(\nu_{z}+g\right)\left(\tan\theta \cos\psi +\frac{\tan\phi \sin\psi }{\cos\theta }\right)+d_{x}$$

(13) $$\ddot{y}=\left(\nu_{z}+g\right)\left(\tan\theta \sin\psi -\frac{\tan\phi \cos\psi }{\cos\theta }\right)+d_{y}$$

where $d_{x}$, $d_{y}$, and $d_{z}$ are the lumped disturbances, including the externally bounded and unmodeled ones. The above equations can be written as follows:

(14) $$\left[\begin{array}{c} \ddot{x}\\ \ddot{y} \end{array}\right]=\left(\nu_{z}+g\right)\left[\begin{array}{cc} \cos\psi & \sin\psi \\ \sin\psi & -\cos\psi \end{array}\right]\left[\begin{array}{c} \tan\theta^{d}\\ \frac{\tan\phi^{d}}{\cos\theta } \end{array}\right]+\left[\begin{array}{c} d_{x}\\ d_{y} \end{array}\right]$$

Defining the virtual control inputs $\theta^{d}$ and $\phi^{d}$,

(15) $$\left[\begin{array}{c} \tan\theta^{d}\\ \frac{\tan\phi^{d}}{\cos\theta } \end{array}\right]=\frac{1}{\left(\nu_{z}+g\right)}{\left[\begin{array}{cc} \cos\psi & \sin\psi \\ \sin\psi & -\cos\psi \end{array}\right]}^{-1}\left[\begin{array}{c} \nu_{x}\\ \nu_{y} \end{array}\right]=\left[\begin{array}{c} \frac{\nu_{x}\cos\psi +\nu_{y}\sin\psi }{\nu_{z}+g}\\ \frac{\nu_{x}\sin\psi -\nu_{y}\cos\psi }{\nu_{z}+g} \end{array}\right]$$

Introducing (15) into (14), we obtain the translational dynamics:

(16) $$\ddot{x}=\nu_{x}+d_{x}$$

(17) $$\ddot{y}=\nu_{y}+d_{y}$$

where $\nu_{x}$ and $\nu_{y}$ are auxiliary controls, which will be defined later.

The error is defined as

(18) $$e\left(t\right)=\left[\begin{array}{c} e_{x}\\ e_{y}\\ e_{z} \end{array}\right]=\left[\begin{array}{c} x-x^{d}\\ y-y^{d}\\ z-z^{d} \end{array}\right]$$

We can rewrite the translational dynamics as follows:

(19) $$\left[\begin{array}{c} \ddot{x}\\ \ddot{y}\\ \ddot{z} \end{array}\right]=\left[\begin{array}{c} \nu_{x}\\ \nu_{y}\\ \nu_{z} \end{array}\right]+\left[\begin{array}{c} d_{x}\\ d_{y}\\ d_{z} \end{array}\right]$$

Sliding surfaces are defined for each auxiliary control of the translational dynamics:

(20) $$s_{x}=\dot{x}+\beta_{x}\left(x-x^{d}\right)$$

(21) $$s_{y}=\dot{y}+\beta_{y}\left(y-y^{d}\right)$$

(22) $$s_{z}=\dot{z}+\beta_{z}\left(z-z^{d}\right)$$

These sliding surfaces are an important stage design of the SMC; these surfaces guarantee the fixed-time convergence of the involved states.

Define the auxiliary controls of the translation dynamics using the SMC of constant exponential coefficients defined in the following expression:

(23) $$\nu_{x}=-\beta_{x}{\dot{e}}_{x}-k_{1x}sign\left(s_{x}\right)-k_{2x}|s_{x}{|}^{\alpha }sign\left(s_{x}\right)-k_{3x}{\left|s_{x}\right|}^{\gamma }sign\left(s_{x}\right)-k_{4x}s_{x}$$

(24) $$\nu_{y}=-\beta_{y}{\dot{e}}_{y}-k_{1y}sign\left(s_{y}\right)-k_{2y}|s_{y}{|}^{\alpha }sign\left(s_{y}\right)-k_{3y}{\left|s_{y}\right|}^{\gamma }sign\left(s_{y}\right)-k_{4y}s_{y}$$

(25) $$\nu_{z}=-\beta_{z}{\dot{e}}_{z}-k_{1z}sign\left(s_{z}\right)-k_{2z}|s_{z}{|}^{\alpha }sign\left(s_{z}\right)-k_{3z}{\left|s_{z}\right|}^{\gamma }sign\left(s_{z}\right)-k_{4z}s_{z}$$

Define the torque control inputs as

(26) $${\tilde{\tau }}_{\phi }=\frac{I_{x}}{l}\tau_{\phi }-\left[\dot{\theta }\dot{\psi }\left(\frac{I_{y}-I_{z}}{I_{x}}\right)\right]$$

(27) $${\tilde{\tau }}_{\theta }=\frac{I_{y}}{l}\tau_{\theta }-\left[\dot{\phi }\dot{\psi }\left(\frac{I_{x}-I_{z}}{I_{y}}\right)\right]$$

(28) $${\tilde{\tau }}_{\psi }=\frac{I_{z}}{l}\tau_{\psi }-\left[\dot{\phi }\dot{\theta }\left(\frac{I_{y}-I_{x}}{I_{z}}\right)\right]$$

Then, we obtain

(29) $$\left[\begin{array}{c} \ddot{\phi }\\ \ddot{\theta }\\ \ddot{\psi } \end{array}\right]=\left[\begin{array}{c} \tau_{\phi }\\ \tau_{\theta }\\ \tau_{\psi } \end{array}\right]+\left[\begin{array}{c} d_{\phi }\\ d_{\theta }\\ d_{\psi } \end{array}\right]$$

where $d_{\phi }$, $d_{\theta }$, and $d_{\psi }$ are lumped perturbations including the external and coupling dynamics. The attitude error is defined as

(30) $$\tilde{\eta }=\eta -\eta^{d}=\left[\begin{array}{c} \phi -\phi^{d}\\ \theta -\theta^{d}\\ \psi -\psi^{d} \end{array}\right]$$

where the sliding surfaces are defined for each auxiliary control of the attitude dynamics:

(31) $$s_{\phi }=\dot{\phi }+\beta_{\phi }\left(\phi -\phi^{d}\right)$$

(32) $$s_{\theta }=\dot{\theta }+\beta_{\theta }\left(\theta -\theta^{d}\right)$$

(33) $$s_{\psi }=\dot{\psi }+\beta_{\psi }\left(\psi -\psi^{d}\right)$$

Define the auxiliary controls of the attitude dynamics using the SMC of constant exponential coefficients in the following expression:

(34) $$\tau_{\phi }=-\beta_{\phi }{\dot{\tilde{\eta }}}_{\phi }-k_{1\phi }sign\left(s_{\phi }\right)-k_{2\phi }|s_{\phi }{|}^{\alpha }sign\left(s_{\phi }\right)-k_{3\phi }{\left|s_{\phi }\right|}^{\gamma }sign\left(s_{\phi }\right)-k_{4\phi }s_{\phi }$$

(35) $$\tau_{\theta }=-\beta_{\theta }{\dot{\tilde{\eta }}}_{\theta }-k_{1\theta }sign\left(s_{\theta }\right)-k_{2\theta }|s_{\theta }{|}^{\alpha }sign\left(s_{\theta }\right)-k_{3\theta }{\left|s_{\theta }\right|}^{\gamma }sign\left(s_{\theta }\right)-k_{4\theta }s_{\theta }$$

(36) $$\tau_{\psi }=-\beta_{\psi }{\dot{\tilde{\eta }}}_{\psi }-k_{1\psi }sign\left(s_{\psi }\right)-k_{2\psi }|s_{\psi }{|}^{\alpha }sign\left(s_{\psi }\right)-k_{3\psi }{\left|s_{\psi }\right|}^{\gamma }sign\left(s_{\psi }\right)-k_{4\psi }s_{\psi }$$

### 4.2. Constant Exponent Coefficient Sliding Mode Control Stability Analysis

Consider the following uncertain nonlinear second-order system:

(37) $$\ddot{z}=-g+\frac{\cos\theta \cos\phi }{m}u+d_{z}$$

and with the sliding surface $\nu_{z}$ proposed for the dynamics of the *z*-axis:

(38) $$s_{z}=\dot{z}+\beta_{z}z$$

with β>0; the controller is proposed as

(39) $$u=\frac{m}{\cos\theta \cos\phi }[g-\beta_{z}z-k_{1z}sign\left(s_{z}\right)-k_{2z}|s_{z}{|}^{\alpha }sign\left(s_{z}\right)-k_{3z}|s_{z}{|}^{\gamma }sign\left(s_{z}\right)-k_{4z}s_{z}]$$

with $k_{1z}>d_{z},k_{2z}>0,k_{3z}\geq 0,k_{4z}\geq 0,\alpha >1$, and 0<γ<1.

**Proposition** **1.** *The closed-loop system (37)–(39) reaches the sliding surface $s_{z}=0$ in fixed-time, satisfying the settling time $T(s_{0})$.*

(40) $$T\left(s_{0}\right)\leq \frac{1}{k_{1z}-\delta }+\frac{1}{k_{2z}\left(\alpha -1\right)}$$

The closed-loop system is globally asymptotically stable.

### 4.3. Stability Proof

Consider the sliding surface $s_{z}$ and the dynamics in $\ddot{z}$ and *u* defined in (39), where *g* represents the force of gravity and *m* the mass of the vehicle. The derivative of the proposed sliding surface ${\dot{s}}_{z}$ is defined as

(41) $${\dot{s}}_{z}=-g+\frac{m}{\cos\theta \cos\phi }u+\beta \dot{z}+d_{z}$$

Introducing (39) into the above, we obtain

(42) $${\dot{s}}_{z}=k_{1z}sign\left(s_{z}\right)+k_{2z}|s_{z}{|}^{\alpha }sign\left(s_{z}\right)+k_{3z}{\left|s_{z}\right|}^{\gamma }sign\left(s_{z}\right)+k_{4z}s_{z}+d_{z}$$

To demonstrate the stability of the system, consider the following Lyapunov candidate function [23]:

(43) $$V\left(x\right)=s_{z}^{2}$$

Then,

(44) $$\dot{V}=-2k_{1z}|s_{z}|-2k_{2z}|s_{z}{|}^{\alpha -1}-2k_{3z}{\left|s_{z}\right|}^{\gamma -1}-2k_{4z}s_{z}^{2}+2d_{z}s_{z}$$

(45) $$\leq -2\left(k_{1z}-\delta_{z}\right)|s_{z}|-2k_{2z}{\left|s_{z}\right|}^{\alpha +1}$$

(46) $$\leq -2\left(k_{1z}-\delta_{z}\right)V{\left(s_{z}\right)}^{\frac{1}{2}}-2k_{2z}V{\left(s_{z}\right)}^{\frac{\alpha +1}{2}}$$

with $\frac{\left(\alpha +1\right)}{2}>1$.

**Remark** **1.** *The function $x\to {\left|x\right|}^{\gamma }sgn\left(s_{z}\right)$ with x(t)∈R,λ>0 and μ>0 such that $\theta =\frac{\lambda }{\left(1+\mu \right)}>1$, with 0<γ<1, which ensures the asymptotic stability of the closed-loop system (37), (38), and (39) towards the origin.*

The stability test is similar to the one in [25] applied to the second-order $\ddot{z}$ dynamics of the quadrotor and also to the other dynamics.

## 5. Results

### 5.1. Simulation Results of the Aerial Vehicle

The simulation for each of the dynamics was developed with different parameters because they are nonlinear and under-actuated, where the perturbation for the translational dynamics is defined as d= [sin(t), sin(t), 2sin(10t)], and the parameters β=1, α=1.5, and γ=0.5 are the same for the translational dynamics and δ= [1.1, 1.1, 2.1]. For the gains $k_{i}$ where i=1,…,4 defined for each independent translational dynamics, one still has $T_{z}\left(s_{0}\right)\leq 1.833s$, and for the other dynamics, being coupled and depending directly on the z dynamics, we define a different settling-time such that $T_{x,y}\left(s_{0}\right)\leq 6s$ with different parameters.

A desired trajectory is defined by

(47) $$q^{d}=\left[\begin{array}{c} \cos(\omega t)\\ -\sin(\omega t)\\ 6 \end{array}\right]$$

where $\omega =\frac{2\pi }{20}$.

**Figure 3:**
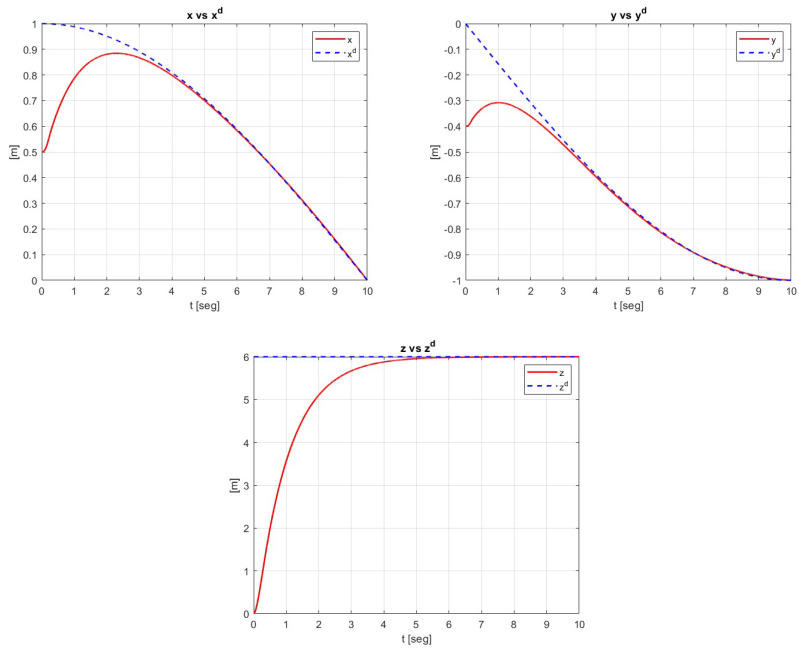
Behavior of the trajectory tracking of the translational dynamics.

The trajectory tracking of the translational dynamics *x*, *y*, and *z* with initial conditions IC= [0.5, −0.4, 0] is shown in Figure 3.

**Figure 4:**
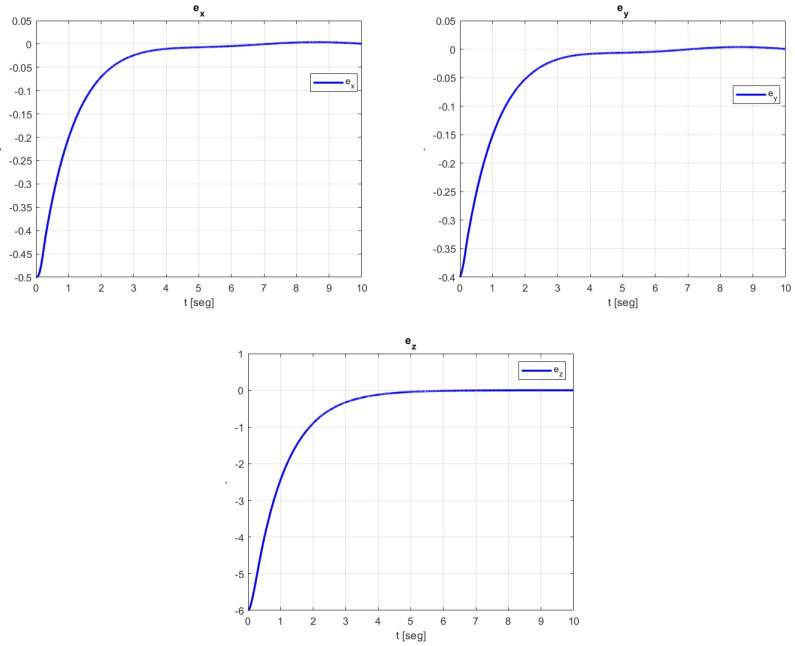
Convergence of tracking errors in the translational dynamics of the quadrotor vehicle.

The tracking errors are shown in Figure 4, and the convergence to zero of the states of the translational dynamics is observed.

**Figure 5:**
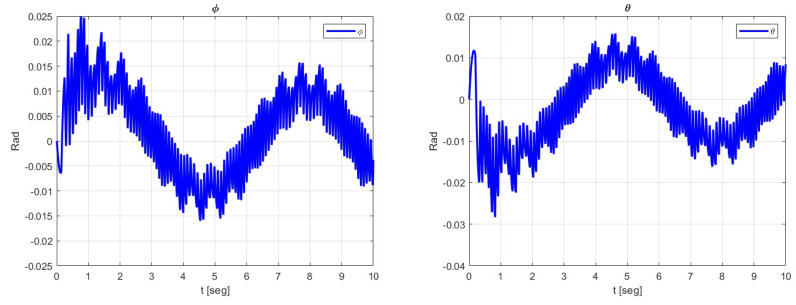

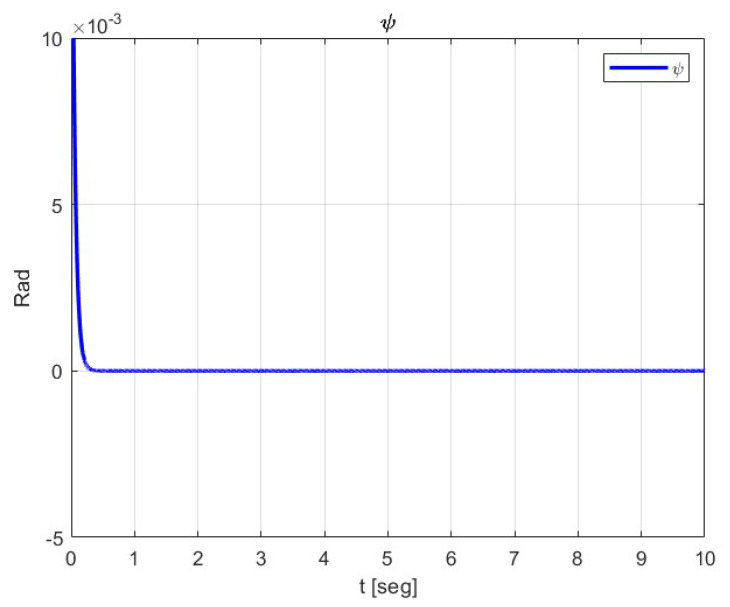
Behavior of the ϕ, θ and ψ dynamics.

The behavior of the trajectory tracking of the attitude dynamics ϕ and θ and ψ is shown in Figure 5.

**Figure 6:**
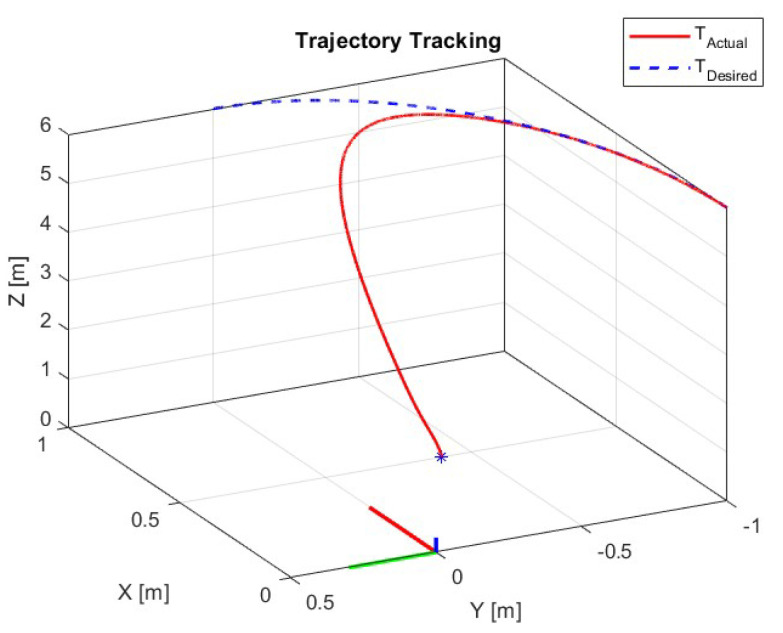
Three dimensional trajectory tracking.

Finally, Figure 6 shows the three-dimensional trajectory tracking carried out by the quadrotor vehicle.

### 5.2. CNN People-Detection Results

**Figure 7:**
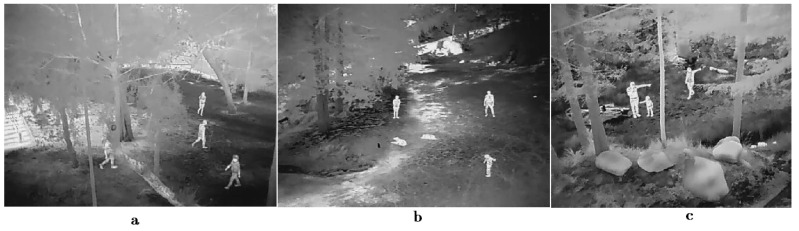
CNN model human detection in a thermal image. (**a**) shows training and validation process example, (**b**) shows no occlusion people example and (**c**) real application.

For the development of the person-detection algorithm, in complex wooded areas using thermal images, it is necessary to consider that, in multiple situations, the object of interest (person) may be occluded by objects in the environment, and this makes detection difficult. This complication requires models that consider many representative features learned from image datasets under the desired detection conditions. The detection model was developed using the generalized thermal imaging dataset of a person when viewed in its entirety, making some first predictions. Then, an extension is made using the thermal imaging data, focused on the characterization of the occlusion condition. With these conditions, we implemented a CNN model with more-representative characteristics for a lost person in forested areas. A FLIR VUO thermal camera was used to capture the videos in the three forested settings shown in Figure 7.

The Figure 7a corresponds the place where video capture was carried out with the camera mounted on the UAV vehicle, capturing multiple videos, which were divided into two parts: one part was used for the training process and the other part for the process of model validation. The Figure 7b corresponds to the videos captured in a controlled way and where there is no occlusion of the persons. With this set of videos, the dataset of images where persons were fully displayed was created. Finally, in Figure 7c, the dataset of images never seen before by the model was generated and the training process was not used. The image dataset was divided into two classes: person and non-person. The first class must contain persons or representative parts in some part of the image, and the second class contains objects in general that represent everything that does not correspond to the representative characteristics of a person.

This architecture was tested for small image datasets and may represent a viable option considering that the detection application is focused on detecting persons, who represent a small portion of the overall image. The models developed so far had complications when defining the architecture because each convolution process reduces the image by a certain proportion. Therefore, very deep networks obtained few representative features of the small portion of the image that made up a person in the image.

### 5.3. Dataset (Thermal Images)

The dataset was made up of images of persons and images that did not contain any person. With the cutouts or sections of images that contained a complete person or with a certain portion of their characteristics occluded by the environment and sections that did not contain any person, which represent the characteristics of the desired environment for inspection, a large number of positive image samples will allow the algorithm to generalize the characteristics of a person for the classification and detection processes.

**Figure 8:**
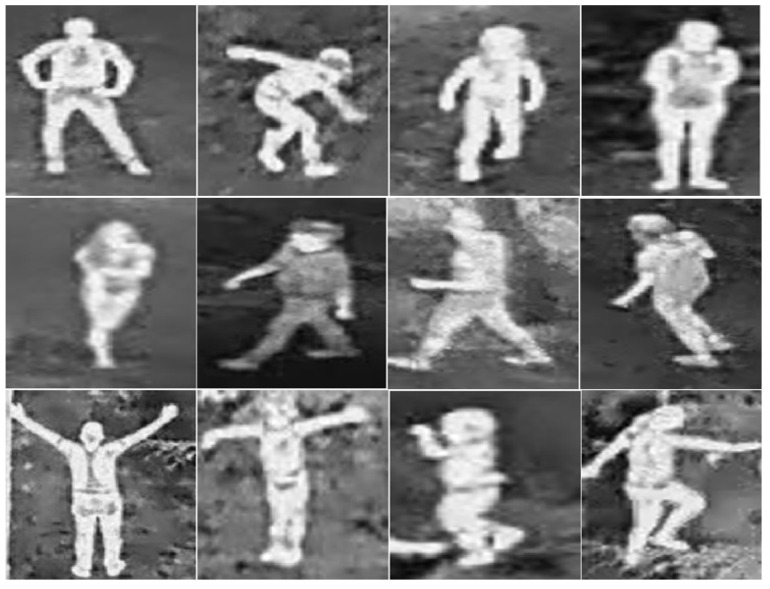
Positive image section containing people.

This procedure was carried out on all the images captured in the three proposed forest scenarios, obtaining a total of 10,000 thermal images of 640 × 520 px (database available in People Thermal Images1, https://doi.org/10.6084/m9.figshare.24473002.v1 (accessed on 18 September 2023)), and the sectioned datasets were made up in the following form:

Complete positive images: 3000 images (150 × 150 px) sectioned as people divided into two sets: 2000 training images and 1000 validation images, see Figure 8.

**Figure 9:**
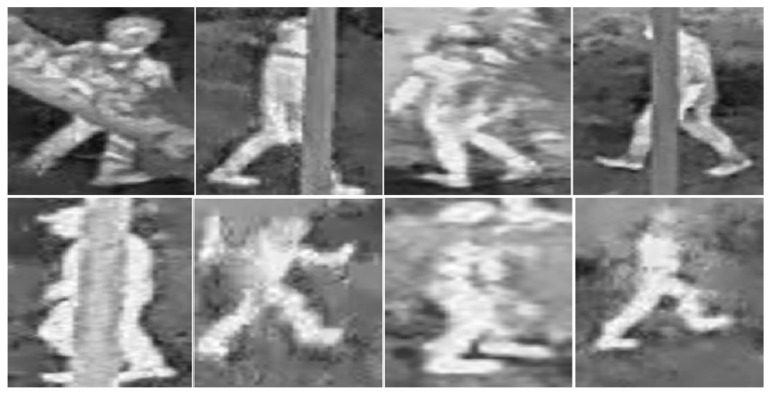
Positive image section with occlusion containing persons.

Positive occlusion images: 2000 images (150 × 150 px) sectioned as persons divided into two sets: 1500 training images and 500 validation images, see Figure 9.

**Figure 10:**
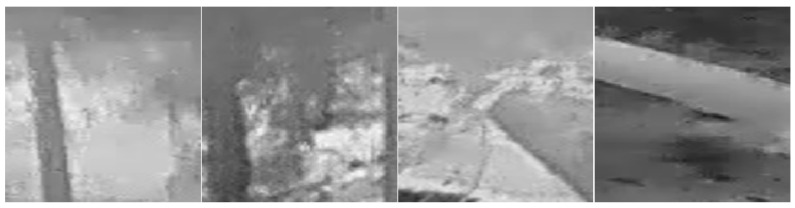
Negative image section containing wooded areas.

Negative images: 5000 images (150 × 150 px) sectioned as persons divided into two sets: 3500 training images and 1500 validation images, see Figure 10.

The forested negative dataset represents all images that did not represent a person, which may be objects, vegetation, or anything that can provide information to the detection model, which accurately generalizes the representative characteristics of a person.

### 5.4. Training the CNN Models

The CNNarchitecture had a sequence of consecutive layers, and this is important to increase the capacity of the network, as well as reduce the size of the feature maps so that they are not too large when we reach the Flatten layer, which will convert them into a one-dimensional vector of values. The procedure starts with input images of size [150 × 150], ends with feature maps of size 2 × 2, followed by a conversion of the 2D array of [2 × 2] to a 1D vector using the Flatten layer. The depth of the feature maps increases progressively in the network (from 64 to 128). This is a pattern that can be observed in all CNNs: it starts with a set dimension image and becomes progressively smaller in order to highlight the most-representative features of the dataset.

Several training processes were carried out with the CNN architecture with the thermal image dataset. This model has a shallow architecture, which requires little computational resources, which allows the proposed codes to be optimized and tested in a more-fluid way. Subsequently, with the tested codes, the architecture with the data extension was used.

For the CNN model, as it only represented a rapid test model, it was trained several times to perform training that was not so precise but that would allow the detection algorithms to be tested in real-time in a fluid and fast way, prior to further training. For the CNN model with a modified architecture, the algorithm trained for 100 epochs, in addition the modification of some of the hyperparameters.

The training processes were developed for 100 epochs using the established dataset considering the partial occlusion of persons in the thermal images. The maximum values of the precision and loss obtained for the two training processes with the best result are observed in Table 1.

**Figure 11:**
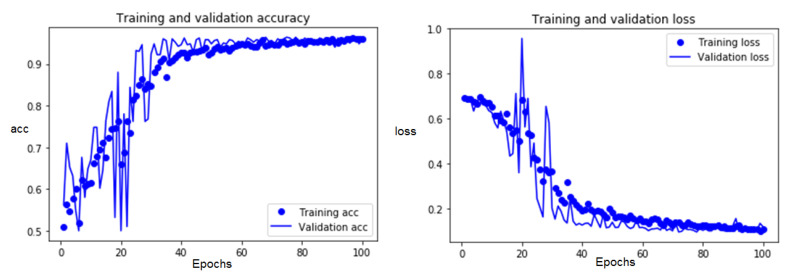
Precision and loss plots of the CNN2 model for the training data extended over 100 epochs.

The CNN1 and CNN2 architectures were the same; the difference was that, in CNN1, it was carried out with data from complete persons, and for CNN2, data in a situation of occlusion were added. The temporal evolution of the maximum values of the precision and loss in the training process for the CNN2 model showed adequate training for the detection of features never seen before by the model, as shown in Figure 11. A progressive training follow-up was observed without separating the estimates; this indicated that the predictions for both the training and validation data had lower loss values.

### 5.5. Detection of a Person in Thermal Images Using Sliding Window

For the evaluation of the performance of the CNN detection model, a sliding window application was used with the weights learned during the training process. In combination with image pyramids and a sliding window process, we implemented an image classifier, which can recognize objects at different scales and locations in the image. These techniques, although they are not a solution for real-time detection, play an absolutely critical role in the performance analysis in object detection and image classification.

**Figure 12:**
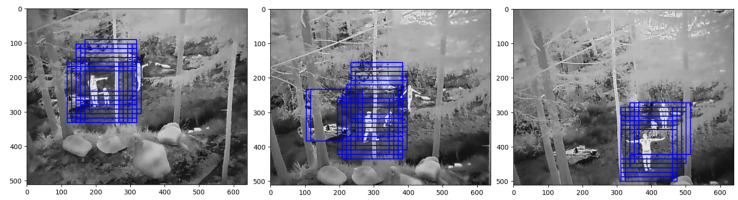
CNN2 model detection using sliding window for images never seen before by the model.

Different complex scenarios of thermal images captured from an aerial perspective, not used in the model training process, were evaluated, obtaining adequate classification results even in situations where the environment is very rugged and there is partial occlusion of the persons shown in Figure 12.

### 5.6. Real-Time CNN Classifier + Haar Cascade

**Figure 13:**
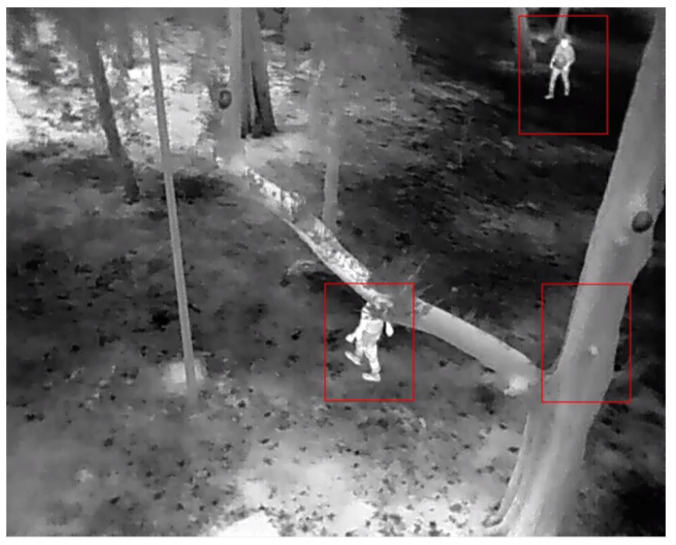
Person detection using Haar cascade model (Viola and Jones) in thermal images.

In a real-time implementation, see Figure 13, the Haar model was run on an NVIDIA Jetson Nano mini-computer with restricted processing resources. The Haar model is a good alternative for testing in a real-time object-detection applications [27].

**Figure 14:**
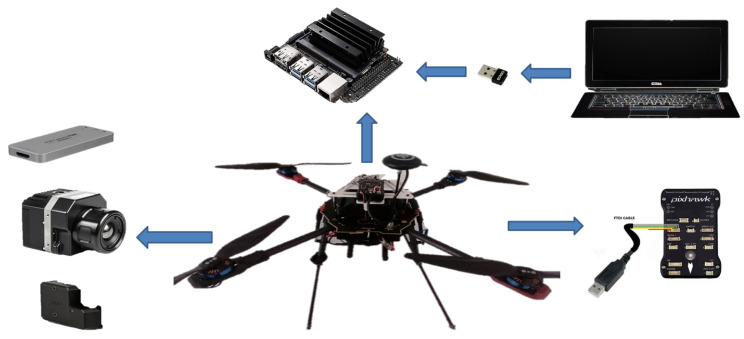
Prototype vehicle used for the evaluation of the CNN model in real-time.

This allowed us to evaluate the CNN model in real-time on board a prototype quadcopter vehicle. In order to test the CNN deep learning model for the detection of persons in thermal images from an aerial perspective and reduce the computational processing requirements, the developed CNN model will only evaluate the selector boxes generated by the Haar model and assign a label for each of the frames. The selector box may represent a person or may represent part of the woodland environment. This implementation will allow the processing to be carried out on board the prototype vehicle. The prototype vehicle used for the CNN model test is shown in Figure 14.

Training the Haar cascade model for our thermal image dataset, we sought to obtain a similar result considering that our dataset was relatively small and, for the Haar classifiers, there are no data extension functions.

The second block of results focused on combining the Haar classifier with a CNN model, which would allow the generation of deep learning predictions on the regions of interest detected by the Haar cascade classifier. This is very useful because, if the Haar classifier generates false positives in its classification process, a second evaluation is still possible, which will define whether it is a person or not and will depend on the prediction values generated by the modified CNN model, allowing us to rule out a large number of false detections.

**Figure 15:**
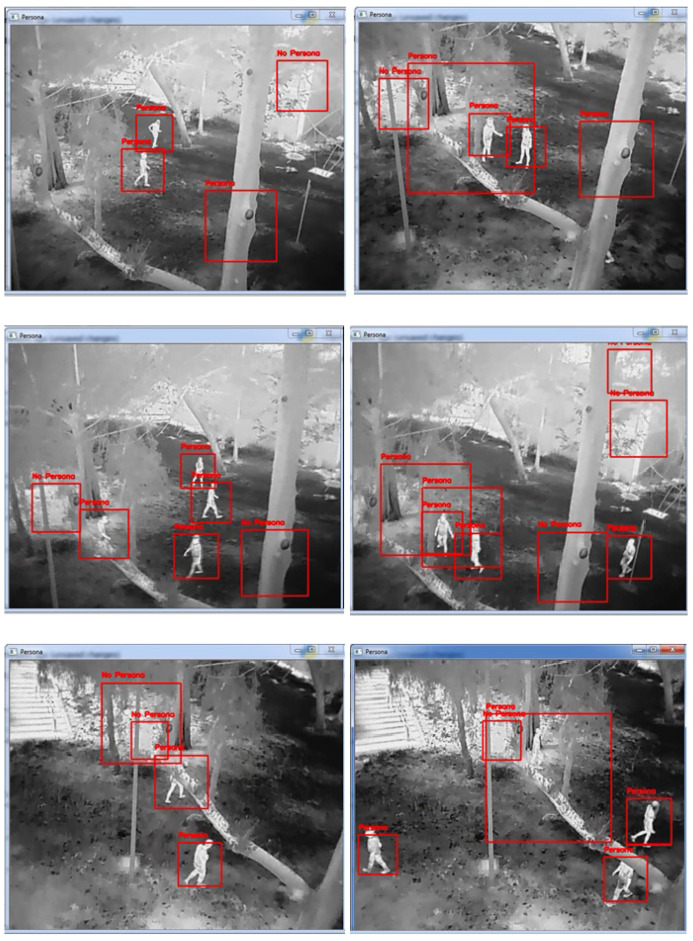
CNN model people detection in real-time for thermal image.

The results in Figure 15 show that, in both images, the regions of interest with exposed persons without occlusion were classified correctly, and for the regions of interest where no persons were found, they were defined with the label “no person”.

## 6. Discussion

The results presented in this work were similar to other publications in the literature. For example, in [28], the authors used several high-performance deep-learning-based object detection algorithms for detecting small objects in aerial thermal images. The detection algorithm was implemented in a workstation computer.

In [29], the authors used a thermal camera using a deep learning model for human detection in low-visibility fire smoke scenarios. Furthermore, Reference [24] presented a new dataset of thermal image sequences for person detection and tracking. Besides, the authors proposed a new framework based on particle filters to track persons in aerial thermal images, using a small computer.

The detection of persons in real-time using thermal images from an aerial perspective is adequate for different circumstances in which the occlusion of certain portions of the features of the human body occur in a wooded area. We observed that false positives may be generated by the Haar cascade model, but were correctly classified by the CNN model with the label of “non-person”. The proposed deep learning CNN model correctly classified most of these positive detections with 99.8% training accuracy for a complete person and 95% training accuracy for an occluded person. Several scenes captured during the test flights with the quadrotor prototype vehicle were presented. The capture of aerial information allowed the creation of a perspective image database that adequately generalized the detection conditions of lost persons in forested areas and was used to train the CNN deep learning model.

## Figures and Tables

**Table 1:** Metrics obtained in the training process of the CNN model.

Metrics	Train_acc	Train_loss	Val_acc	Val_loss
CNN1	0.98 (98%)	0.00247	0.96 (96%)	0.00183
CNN2	0.998 (99.8%)	0.00093	0.98 (98%)	0.00032

## Data Availability

People thermal images database available in https://doi.org/10.6084/m9.figshare.24473002.v1 (accessed on 18 September 2023).
